# TASKA: A modular task management system to support health research studies

**DOI:** 10.1186/s12911-019-0844-6

**Published:** 2019-07-02

**Authors:** João Rafael Almeida, Rosa Gini, Giuseppe Roberto, Peter Rijnbeek, José Luís Oliveira

**Affiliations:** 1 University of Aveiro, DETI/IEETA, 3810-193 Aveiro, Portugal; 2 Agenzia Regionale di Sanit della Toscana, Florence, Italy; 3 Erasmus MC, Rotterdam, Netherlands

**Keywords:** Clinical studies, Task management, Workflow management

## Abstract

**Background:**

Many healthcare databases have been routinely collected over the past decades, to support clinical practice and administrative services. However, their secondary use for research is often hindered by restricted governance rules. Furthermore, health research studies typically involve many participants with complementary roles and responsibilities which require proper process management.

**Results:**

From a wide set of requirements collected from European clinical studies, we developed TASKA, a task/workflow management system that helps to cope with the socio-technical issues arising when dealing with multidisciplinary and multi-setting clinical studies. The system is based on a two-layered architecture: 1) the backend engine, which follows a micro-kernel pattern, for extensibility, and RESTful web services, for decoupling from the web clients; 2) and the client, entirely developed in ReactJS, allowing the construction and management of studies through a graphical interface. TASKA is a GNU GPL open source project, accessible at https://github.com/bioinformatics-ua/taska. A demo version is also available at https://bioinformatics.ua.pt/taska.

**Conclusions:**

The system is currently used to support feasibility studies across several institutions and countries, in the context of the European Medical Information Framework (EMIF) project. The tool was shown to simplify the set-up of health studies, the management of participants and their roles, as well as the overall governance process.

## Background

Health research studies aim to develop new treatments, improve the outcomes of treatment, allow public health and pharmaceutical surveillance, monitor health crises, increase the understanding of diseases and develop guidelines for best clinical practices [1].

These studies can be divided into two broad categories: experimental studies (e.g., randomised controlled trials) and observational studies (e.g., cohort studies, case-control studies). In a preliminary study, the researcher has some intervention, e.g., through the administration of a new drug [2], while in an observational study researchers observe and collect information without intervention. These studies are multi-step processes formed of three main phases: the design, carrying out, and analysis of the study. To ensure high quality, each of these phases must be carefully planned, which usually involves a multi-disciplinary team of statisticians, methodologists, clinical researchers and laboratory scientists, among others [3].

To gain access to clinical digital data, researchers have to deal with complex processes that include study submission, governance approval, data harmonisation, data extraction and many other tasks [4–6]. This process can be simplified by using task and workflow management systems. Furthermore, they can also be used to streamline all the processes associated with a health research study.

Scientific workflow systems allow the composition and execution of a set of computational processes, in cascade, and over a distributed environment. Some of these systems may be used to simplify research studies [7, 8].

Taverna is a scientific workflow management system, available as a suite of open-source tools, which is used to facilitate computer simulation of repeatable scientific experiments. It can be executed in a self-hosted server or as a desktop client. The system follows a service-oriented architecture (SOA) approach, which makes the various web interfaces available for external software integration. It is a highly specialised and widely adopted platform, but is less suited to the diverse set of steps in a typical health research study [9]. Galaxy is another popular scientific workflow management system. This cloud-based platform is oriented to facilitate the execution of computational processes over biomedical datasets. The main purpose of the system is to be easy to use by people without technological knowledge, to allow reproducibility of experiments and to facilitate the sharing of results. Galaxy integrates external tools into a user-friendly web interface, allowing the linear cascading of processes and providing, at the same time, access to several bioinformatics datasets. It allows collaborative discussion of results and studies’ replication, but the system architecture is mainly oriented to computational process pipelines [10].

Besides these two scientific-oriented applications, there are several workflow management systems with a broader scope. However, most of them are commercial and do not allow integration with other external systems. Wrike, for instance, is a collaborative platform, where users can assign tasks and track deadlines and schedules. It follows the workflow model and allows integration with document management solutions. Asana is another cloud-based solution, targeted at project and task management, which can be helpful for teams that handle multiple projects at the same time.

Whenever integration within another system is the main requirement [11], a workflow engine may be a good solution. This kind of engine does not offer a ready-to-use solution, but only the base blocks to build the final system. Although this brings the obvious disadvantage of having to develop the end-user application, it also brings several advantages, mainly due to the flexibility to integrate other software modules.

FireWorks is another open-source project, which is focused on the management and execution of scientific workflows [12]. It provides integration with other task queuing platforms, but is focused mostly on parallel work execution and job scripting and processing. jBPM is an open-source business process management suite, which runs as a Java EE application to execute repeatable workflows [13]. The system supports multi-user collaboration, using groups of users, but its configuration is rather complex for users without technical skills. The Activiti BPMN platform is a lightweight engine focused on open source Business Process Management (BPM), targeted at the needs of business professionals, developers and system administrators. This platform allows complex repeatable workflows with different kinds of tasks, but with only one assignee at a time, even though it enables reassignments in the middle of a process.

These task- and workflow-oriented systems have distinct features and goals, and there is a need to combine some key aspects of both systems, namely asynchronous manual/automatic tasks and the integration with external tools. Furthermore, existing workflow engines do not support multi- user features such as users’ collaboration over the same workflow, discussion of results and workflow sharing between different users.

In this paper, we present TASKA, a task/workflow management system that was built as a modular web platform to facilitate studies’ execution, team coordination, task scheduling, and researcher collaboration. In the following sections, we will present the main functionalities of this system, focusing on the end-user perspective. To evaluate its potential, we describe a use case which aims to estimate the prevalence and incidence of acute myocardial infarction in a set of heterogeneous sources of observational health data. The system was developed in the context of European Medical Information Framework (EMIF), a European project that aims to create a common technical and governance framework to facilitate the reuse of health data. TASKA is publicly available at https://bioinformatics.ua.pt/taska.

## Implementation

### System Requirements

The EMIF is an EU project that aims to facilitate the reuse and exploitation of patient-level data, from different electronic health record systems and cohorts. The EMIF Platform, a key result of EMIF, intends to be an integrated system to allow researchers to browse information at three different conceptual levels [14]. The first level allows browsing a catalogue containing database fingerprints, i.e., a general characterisation of the databases [15], the second level provides sets of aggregated data from several databases and the third level allows drilling down to the level of individual patients in those databases.

Conducting a multi-centre study generally implies dealing with multiple organisational issues, from access control policies to the data analyses [16]. In this context, a task management solution is the key to manage all the steps and responsibilities in each study. However, as previously discussed, it is hard to find a solution that combines the potential of a task execution system with a workflow management system, i.e., allows the definition of workflows that mix computational processes with human-oriented tasks.

The task and workflow management system needs to be easy to use, highly modular, and easy to extend with new functionalities, to ensure wide adoption. Together with a users’ group in EMIF, we defined the functional requirements, such as:

- Users may assume two distinct roles: a) Study manager, governing the execution of a workflow; and b) Task assignee, responsible for parts of the workflow execution;
- Tasks should be defined using several templates, namely *manual*, *questionnaire*, and *service*; they may be assigned to different actors, with distinct deadlines and requirements;
- Each workflow needs to combine any sequence of tasks, pipelining the previous outputs to the next tasks’ inputs; the workflow manager must be able to follow and share its execution;
- The system needs to notify users about task deadlines and progress; Moreover, users should be able to give feedback in a interactive way;
- The system should include backoffice facilities to manage users, activities and roles, using role-based access control policies (RBAC).

### Software architecture

**Fig. 1:**
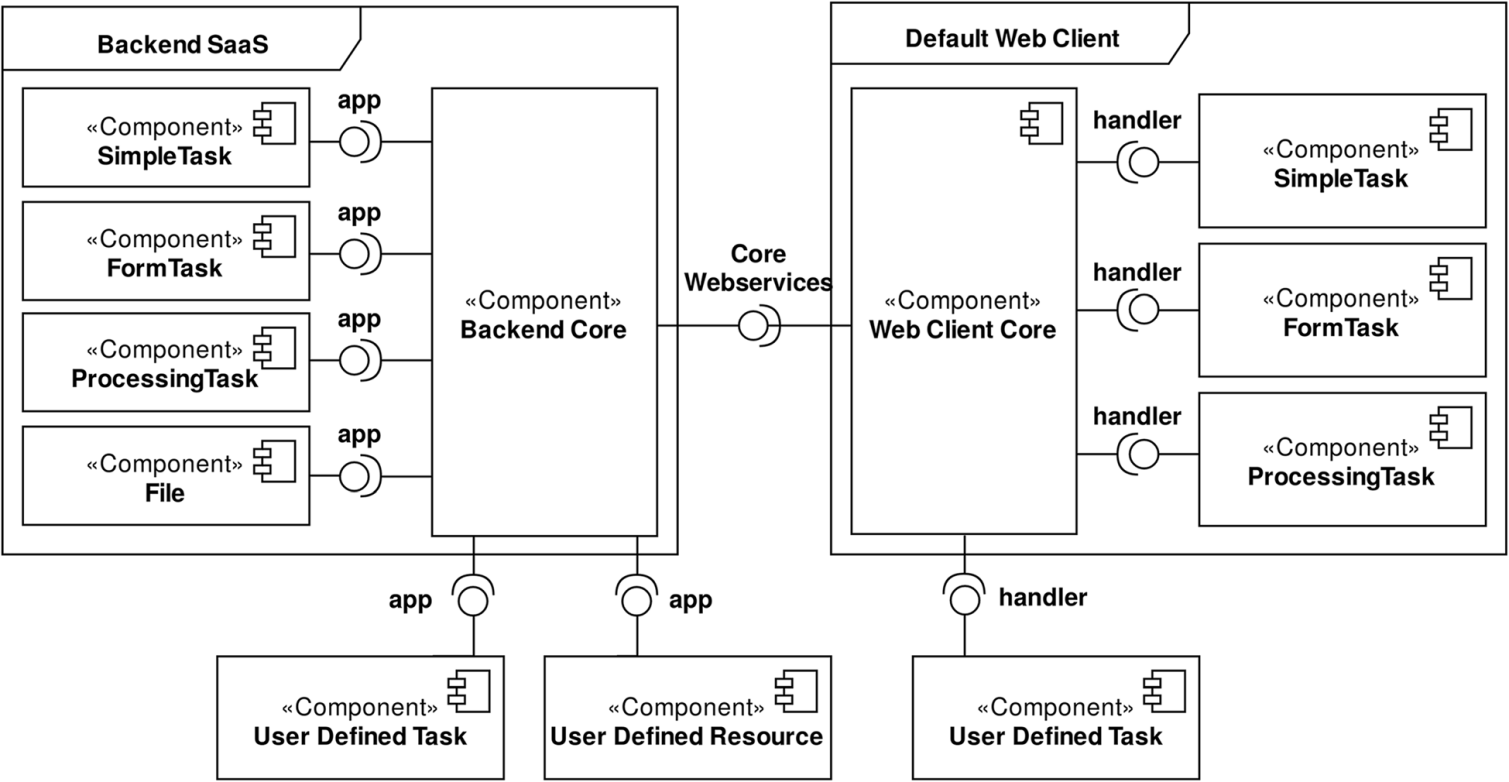
TASKA general architecture

To address the initial requirements, we developed the task/workflow management system in a two-layered architecture. Figure 1 shows the backend engine (*Backend Core*), which ensures the application’s business logic based on Django, a Python web framework. Also, it shows the frontend client (*Web Client Core*), built upon ReactJS, a JavaScript framework, which relies on the backend web services. The backend was entirely developed as RESTful web services, to simplify its integration with the TASKA client, but also with other applications. For the engine, we followed a micro-kernel pattern [17, 18] to allow easy incorporation of new types of tasks and components.

**Fig. 2:**
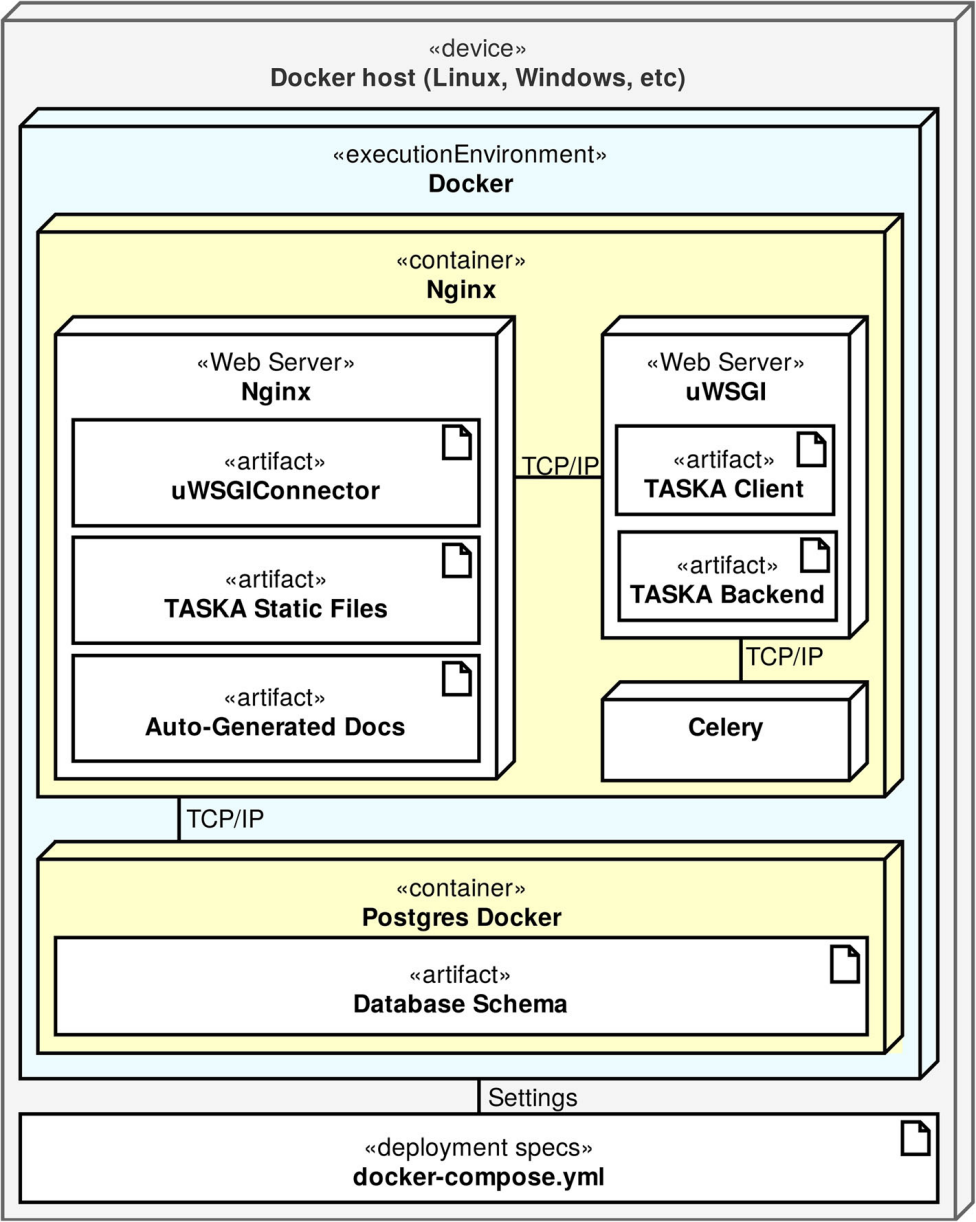
Deployment organization

To simplify the deployment of these components, we used the Docker virtualisation technology (Fig. 2). The docker-compose tool is used to define multi-containers, connections, and all necessary parameters.

TASKA services were developed following the characteristics and requirements of three complementary entities: a) Tasks, i.e., what to do; b) Workflows, how to do; and c) Users, who will do. These elements’ functionalities allow a team to conduct any kind of study in TASKA.

### Tasks

A task is the basic information unit in the system. Each task is organised in three main components: the input, e.g., the data files that are necessary for the task; the definition, i.e., what needs to be done in this task; and the output, the results of the assignment.

To address all the foreseen scenarios for our task/workflow management system, we created three distinct types of task:

1. a *Simple task*, in which the description provides the instructions about what must be performed;
2. a *Form task*, which allows the construction of a simple online questionnaire (text, multiple choices, etc.) that needs to be completed by each assignee. Each form is created using a drag-and-drop graphical user interface;
3. a *Processing task*, which allows automatic execution of RESTful services provided by external systems. The task definition consists of describing the web-services end-point, and also the parameters that will be used.

### Workflows

**Fig. 3:**
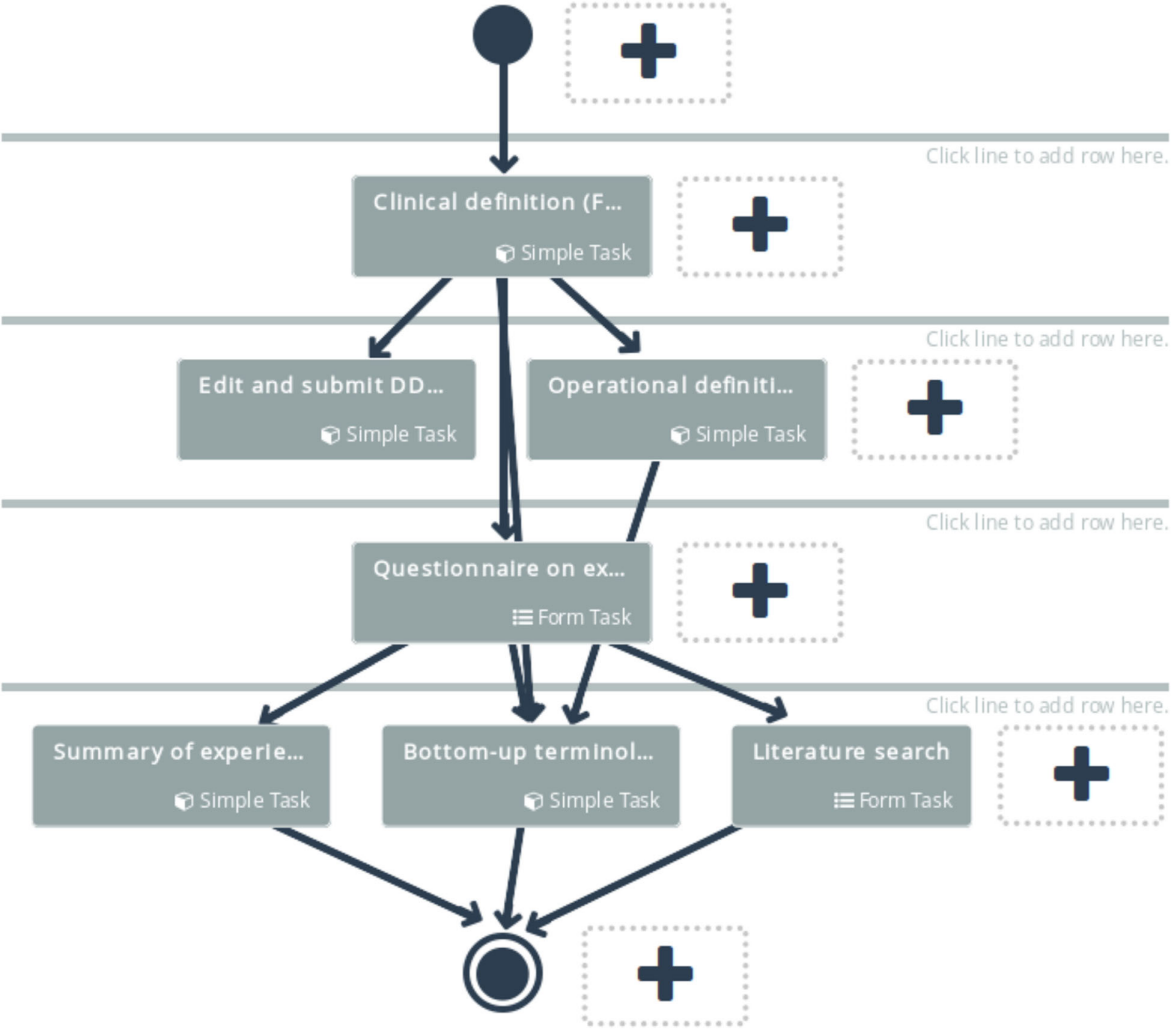
Study template creation

A workflow consists of combining a set of tasks in a hierarchical order (Fig. 3). The workflow begins with a single task, and then, in the following level, it can proceed with one or more parallel tasks. This process, distribution or aggregation, can be repeated up to the last layer, where the final task will collect the final results of the workflow. The web interface can create each workflow in a user-friendly manner. Each box, representing a task, can be configured according to its type. The dependencies between tasks are described as connection lines, which can also be added/removed through the web interface.

When completed, the resulting workflow must be saved to serve as a template for workflow executions, i.e., conducting a clinical study, coordinating teamwork, or similar processes. Each execution of the template is an independent process, having a particular set of participants and deadlines. This means that the same template can be reused to manage several studies over time.

### Users and Studies

**Fig. 4:**
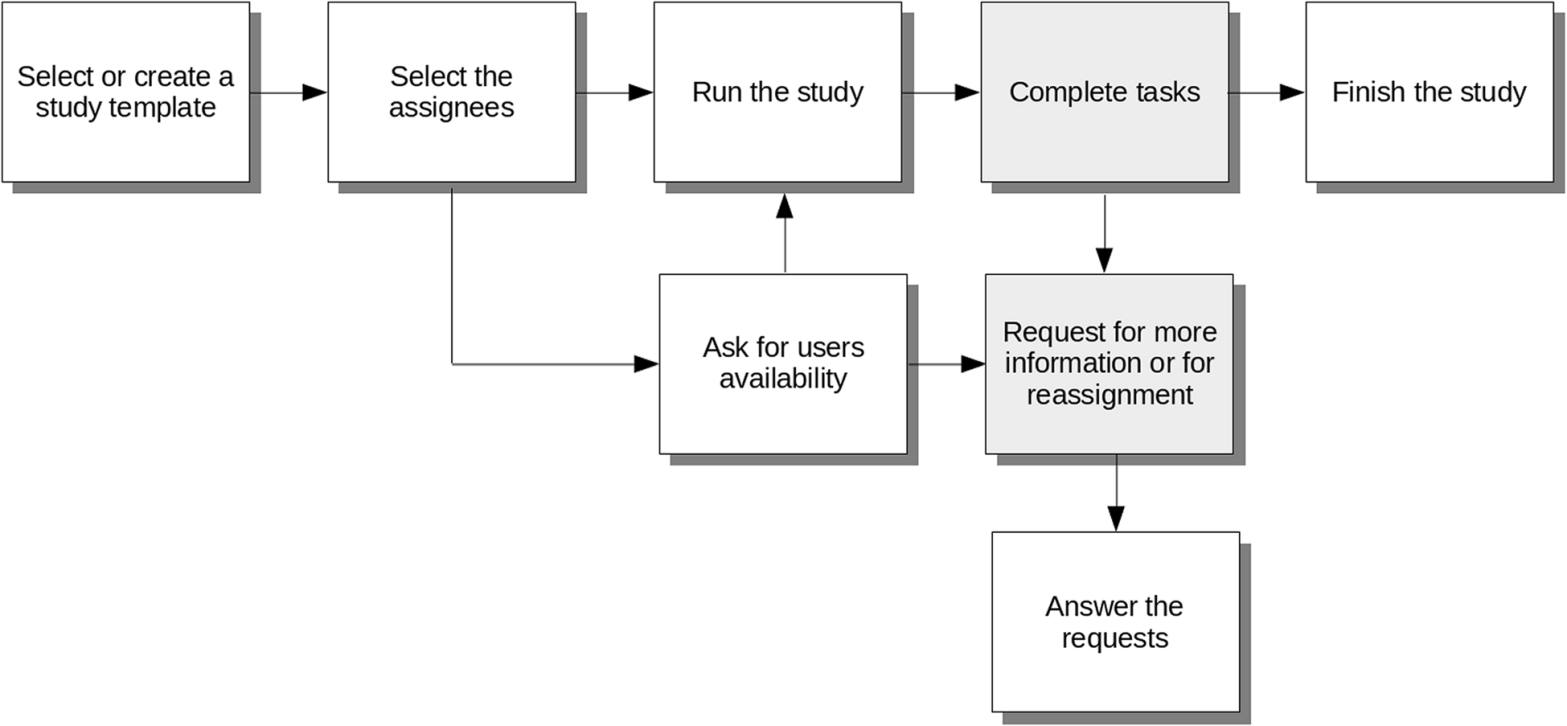
Users workflow - for the study manager (in white boxes), and for the assignee (in gray)

The web client can be described from two different user perspectives: the study manager’s perspective and the task assignee’s perspective. Figure 4 presents the main phases of each study and the role of each user in this pipeline. The components displayed in grey refer to the actions taken by the assignees, and the remainder are the responsibility of the SM.

#### Study manager

The SM role is assumed automatically by any user that creates a study template and decides to execute the workflow within a team of other users (acting here as assignees). Besides defining and coordinating the study pipeline, the SM is responsible for task assignment, scheduling management, and results compilation at the end of the study.

Each user may create study templates, which work as a model that can be used to initiate a study. This template is kept private, in the user workspace, unless they decide to share it with other platform users. The latter may then clone or reuse, but not change the original template. To start a study, the user may create a new template, or select an existing one from the available list. Then, the user, now in an SM role, needs to choose the users that will be involved in the study. In this phase, the SM can also activate some general reminders that will be sent to the task assignees, before and after each task deadline.

The next step is to assign users to tasks. Each task can be attributed to multiple users, and each user can be assigned to several tasks. In the workflow, the SM may ensure that a task is completed only when all assignees finalise the assignment. After this configuration step, i.e., all the tasks have been assigned, the SM must decide if the study should start right away, or if each assignee should confirm their availability to participate in the study. In the latter scenario, if some assignees are not available, the SM can remove or reassign them in the corresponding task(s). This reassignment can also be performed at any time, even after the study has started.

During the study execution, the SM can still reconfigure a task. For instance, if one of the assignees does not complete a task, the SM may remove the user from the study, to avoid delaying the entire process. After task completion, the SM may ask for refinements, which imply doing the task again.

**Fig. 5:**
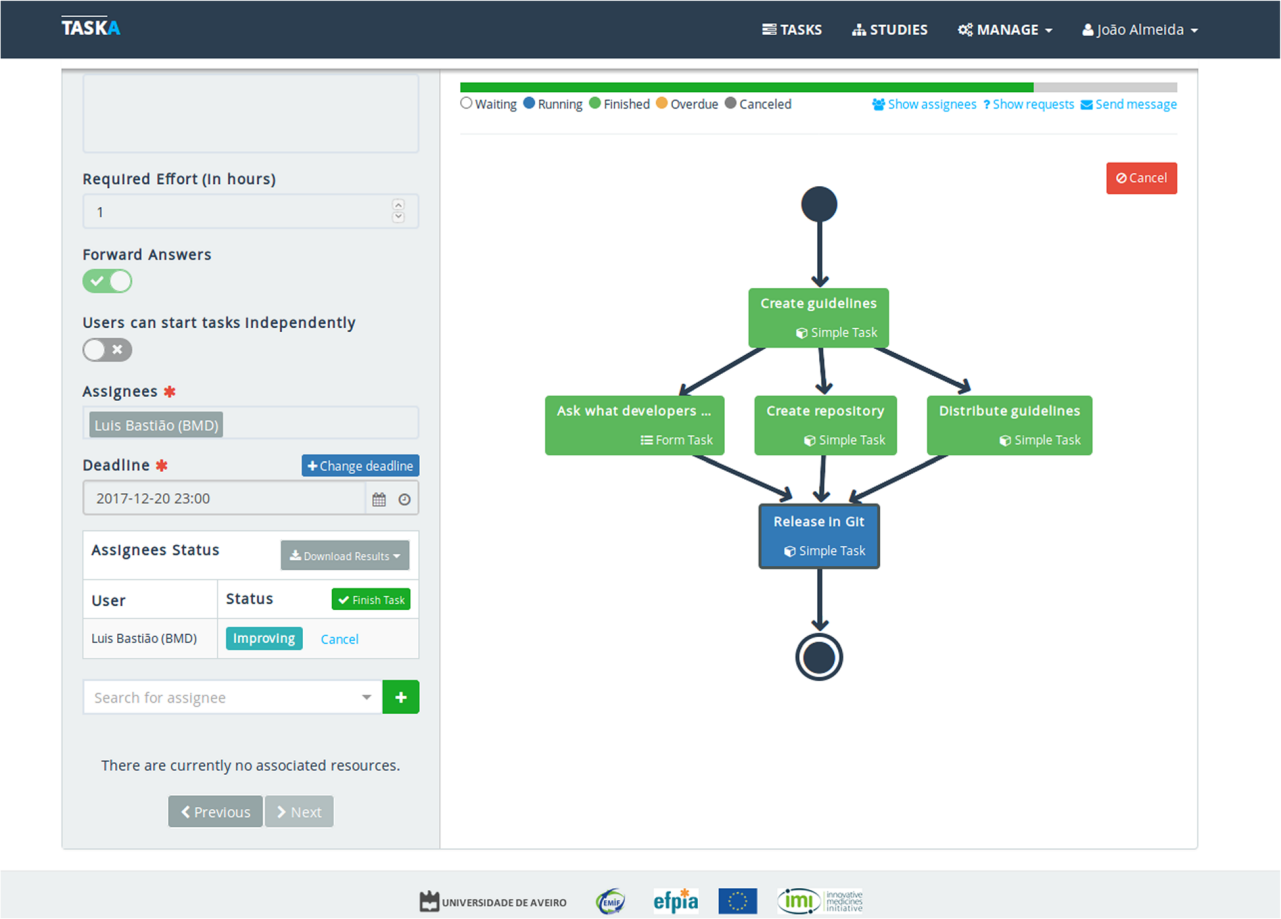
A study representation

Figure 5 presents a running study, where it is clear which tasks were already completed and which remain to be done. This study manager view can also be shared with the study team.

#### Assignees

**Fig. 6:**
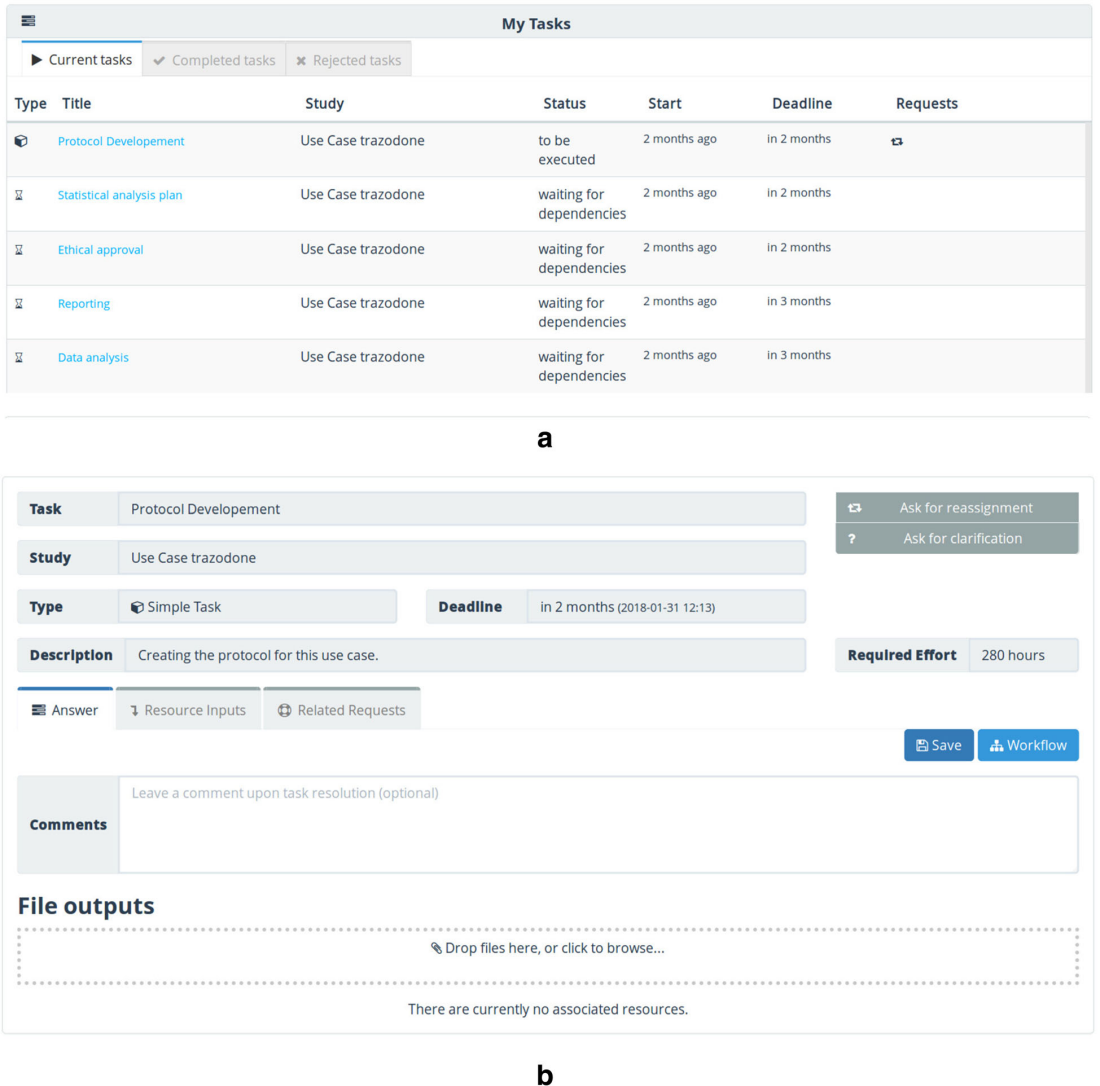
Example of activities in the assignees’ workspace: **a**) list of current tasks. **b**) task details

To build the assignee perspective, we were inspired by the typical email interfaces, i.e., a workspace with a list of requests (tasks), classified according to their priority: pending, solved and rejected. This workspace is the starting point of user activities (Fig. 6).

TASKA sends an email to all the assignees, whenever they are invited to, or included in, a study. Besides the email interactions, all the requests are listed in the user workspace, where they can accept, ask for further details, or complete each task.

This question and answer interaction, between the assignee and the SM, can be kept private or public for the whole team, so that the other participants in the task can see the discussion, avoiding duplicate issues. This feature allows information exchange and user collaboration inside the workspace, keeping all that history aggregated to the task.

In some situations, the assignee needs to improve the completed task. Therefore, they need to use that feature to get the SM’s permission to refine the task result.

## Results

The main motivation for TASKA development was to improve the support of clinical studies, namely when several data filtering and harmonisation processes need to be performed separately by multiple users. Despite having developed a very generic solution, which can be used both as a server-side engine and as a full client-server application, the focus of EMIF is on carrying out clinical studies.

During the systems development, several iterative cycles were conducted, joining developers and users, a methodology adopted inside the EMIF project. In each cycle, a demo was provided for testing and to gather users feedback. This continuous process led to the current version of Taska.

TASKA was an essential tool in a clinical case which aims to estimate the prevalence and incidence of acute myocardial infarction in a set of heterogeneous sources of observational health data collaborating in the EMIF project. These data sources differ in terms of database structure, contents, reasons for recording, language, coding terminologies and healthcare system organisation. As a consequence, each of them may have different strengths and limitations, regarding the identification of a study variable of interest [19]. Data source-tailored case-finding algorithms need to be identified, tested and chosen [20] through an expedited procedure [21]. With this goal, a structuring process was defined, named Data Derivation Workflow (DDW), which was first tested in the case of Type 2 Diabetes Mellitus [22].

The study plan started by defining the number of interactions between the different users, establishing all the dependencies among tasks, and then, the DDW was implemented in TASKA. Eleven users were involved in this study: one SM, with governance responsibility for the entire process; one principal investigator (PI), with scientific leadership; eight data custodians (DC), responsible for ensuring data access and knowledge of the strengths and limitations of their data source; and one terminology mapper (TM), responsible for handling the different terminologies (coding systems and natural languages) used in the data sources.

The study was structured in 13 tasks, each with detailed instructions loaded in the system. Dependencies between tasks were described, in such a way that independent tasks could be executed in parallel. The output of each task was automatically included as an input resource for the dependent tasks. When all the dependencies were solved, users were notified that they could start the dependent task, with an email directing them to the file of the task in their TASKA account. By following the instructions, they extracted and transformed their data, and uploaded the resulting datasets in a protected environment external to TASKA. When a DC had completed the data extraction task, they entered TASKA and manually recorded that the data was available in the protected environment. The PI was notified by TASKA and was invited to access the protected environment to execute the data analysis task.

The case study was the identification of Acute Myocardial Infarction (AMI) in eight European healthcare data sources. The study is compliant with the Code of Conduct of the European network of Centres for Pharmacoepidemiology and Pharmacovigilance, and was registered in the EU PAS Register of studies [23].

The experience from the reported use case demonstrated the potential of TASKA in the semi-automatic management of complex workflows, for the execution of multi-national, multi-database studies for healthcare research.

## Conclusions

Conducting a multi-centre clinical study typically implies dealing with multiple sociotechnical issues, from access control policies to the data analyses. The coordination of all these processes is a complex task, which involves, among others, negotiation, data extraction and data analyses. Managing and keeping track of all these interactions is the main concern for researchers.

TASKA is a simple and intuitive task/workflow management system that can help overcome this complexity, by allowing studies’ execution, team management, and a registry of actions. The system is being used in the EMIF EU project, at a European scale, helping to simplify the participants’ tasks and reducing the time spent on the execution of biomedical research studies.

## Data Availability

No real data was used. Regarding materials, a demo version of TASKA is available at https://bioinformatics.ua.pt/taska. The source code is publicly available at https://github.com/bioinformatics-ua/taska.

## Abbreviations

AMI: Acute Myocardial Infarction
BPM: Business Process Management
DC: Data Custodian
DDW: Data Derivation Workflow
EMIF: European Medical Information Framework
HTML5: Hypertext Markup Language
PI: Principal Investigator
RBAC: Role-based Access Control
RESTful: Representational State Transfer
SM: Study Manager
SOA: Service-Oriented Architecture
TM: Terminology Mapper
